# A Multiscale Model to Investigate Circadian Rhythmicity of Pacemaker Neurons in the Suprachiasmatic Nucleus

**DOI:** 10.1371/journal.pcbi.1000706

**Published:** 2010-03-12

**Authors:** Christina Vasalou, Michael A. Henson

**Affiliations:** Department of Chemical Engineering, University of Massachusetts, Amherst, Massachusetts, United States of America; University College London, United Kingdom

## Abstract

The suprachiasmatic nucleus (SCN) of the hypothalamus is a multicellular system that drives daily rhythms in mammalian behavior and physiology. Although the gene regulatory network that produces daily oscillations within individual neurons is well characterized, less is known about the electrophysiology of the SCN cells and how firing rate correlates with circadian gene expression. We developed a firing rate code model to incorporate known electrophysiological properties of SCN pacemaker cells, including circadian dependent changes in membrane voltage and ion conductances. Calcium dynamics were included in the model as the putative link between electrical firing and gene expression. Individual ion currents exhibited oscillatory patterns matching experimental data both in current levels and phase relationships. VIP and GABA neurotransmitters, which encode synaptic signals across the SCN, were found to play critical roles in daily oscillations of membrane excitability and gene expression. Blocking various mechanisms of intracellular calcium accumulation by simulated pharmacological agents (nimodipine, IP3- and ryanodine-blockers) reproduced experimentally observed trends in firing rate dynamics and core-clock gene transcription. The intracellular calcium concentration was shown to regulate diverse circadian processes such as firing frequency, gene expression and system periodicity. The model predicted a direct relationship between firing frequency and gene expression amplitudes, demonstrated the importance of intracellular pathways for single cell behavior and provided a novel multiscale framework which captured characteristics of the SCN at both the electrophysiological and gene regulatory levels.

## Author Summary

Circadian rhythms are ∼24 hour cycles in biochemical, physiological and behavioral processes observed in a diverse range of organisms including *Cyanobacteria*, *Neurospora*, *Drosophila*, mice and humans. In mammals, the dominant circadian pacemaker that drives daily rhythms is located in the suprachiasmatic nucleus (SCN) of the hypothalamus. The SCN is composed of a highly connected network of ∼20,000 neurons. Within each individual SCN neuron core clock genes and proteins interact through intertwined regulatory loops to generate circadian oscillations on the molecular level. These neurons express daily rhythmicity in their firing frequency and other electrophysiological properties. The mechanisms by which the core clock produces synchronized rhythms in neural firing and gene expression are postulated to involve intracellular calcium, a second messenger that regulates many cellular processes. The interaction between the various clock components however remains unknown. In this paper, we present a single cell model that incorporates the circadian gene regulatory pathway, cellular electrophysiological properties, and cytosolic calcium dynamics. Our results suggest a possible system architecture that accounts for the robustness of the circadian clock at the single cell level. Our simulations predict a dual role for intracellular pathways instigated by intracellular calcium and VIP: maintaining the periodicity and amplitude of the core clock genes as well as the firing frequency oscillations.

## Introduction

In mammals many physiological and behavioral responses are subject to internal time-keeping mechanisms or biological clocks. Daily rhythms are generated by an internal, self-sustained oscillator located in the suprachiasmatic nucleus (SCN) of the hypothalamus. The SCN produces autonomous 24h cycles in gene expression and firing frequency from the synchronization of multiple individual oscillatory signals across the network [1]. The single cell gene regulatory mechanism involves a number of interlocking positive and negative feedback loops in which the *Period* (*Per*) gene occupies the central position [2]. The circadian modulation of neural firing affects a number of electrophysiological properties of the cell membrane which also fluctuate over the course of the day [3].

*In vitro* studies of SCN slices and cultures have demonstrated diurnal modulation of neural firing [4], resting potential [5] and membrane resistance [6], as well as daily oscillations in a number of ionic currents that include the fast delayed rectifier potassium [7], L-type calcium [6] and the large-conductance Ca^2+^-activated potassium [8],[9] channels. Although individual SCN neurons contain molecular feedback loops that drive such rhythms, membrane excitability and synaptic transmission also play significant roles in generating daily oscillations. Experimental studies in *Drosophila* have demonstrated the dependence of core clock oscillations on electrical activity, as electrical silencing resulted in abolishment of circadian oscillations of the free-running molecular clock [10]. In mammalian organisms a direct association between membrane excitability and core-clock rhythms has also been reported in multiple studies, providing evidence for a positive correlation between *Per* gene transcription and neural spike frequency output [11]–[13]. For example, activation of GABA_A_ receptors via muscimol enhanced inhibitory postsynaptic currents (IPSCs) leads to lower firing rates [14],[15] and suppression of *Per1* mRNA [12]. Another example involves mice deficient in vasoactive intenstinal peptide (VIP) receptors known to display lower amplitude oscillations of both core clock genes [16] and neural firing [17].

The mechanisms by which the single cells produce synchronized rhythms in neural firing, gene expression and neuropeptide secretion are postulated to involve intracellular second messengers [18]. A candidate second messenger that regulates diverse cellular processes is intracellular calcium. Cytosolic calcium is known to oscillate over the course of the day preceding rhythms in multiple-unit-activity (MUA) recordings by a mean phase of ∼4.5 hr [4]. Variations in intracellular calcium concentrations have been demonstrated to induce *Per1* gene expression by activating the Ca^2+^/calmodulin dependent kinase, which in turn phosphorylates the cAMP-response-element binding (CREB) protein [19]. Reduced Ca^2+^concentrations have been shown to abolish daily *Per1* mRNA oscillations in SCN slices [20]. Cytosolic Ca^2+^rhythms also affect neural firing frequency, as dampening of Ca^2+^ oscillations via blockade of calcium release from ryanodine-sensitive pools results in decreased firing activity [4],[6].

To our knowledge, detailed cell models with molecular descriptions of gene expression and neural firing coupled by intracellular signaling pathways are not currently available for any circadian system. In a recent study (Sim and Forger 2007), a Hodgkin-Huxley type model of SCN neurons was developed and shown to reproduce a significant amount of experimentally observed electrophysiological behavior on a *millisecond timescale*. While this study facilitated the formulation of our electrophysiology model by providing guidelines for the mathematical representation of a number of relevant ionic currents, our modeling study was distinct due to its focus on the *circadian timescale*. In addition to incorporating single-cell electrophysiological properties, our model has accounted for circadian rhythmicity by coupling electrophysiology to daily oscillations in core-clock gene expression and calcium dynamics. The objective of the present study was to model couplings between the circadian gene-regulatory pathway, cellular electrophysiology and cytosolic calcium dynamics to evaluate the role of extracellular synaptic stimuli on firing rate behavior over a circadian timescale. The role of distinct intracellular pathways as well as the directionality of information flow along the network nodes was evaluated by analyzing single cell model behavior following the introduction of various external stimuli. Calcium dynamics, adapted from a published model [21], included the contributions of IP3- and ryanodine stores as well as the flux of Ca^2+^ in and out of the cell membrane.

Our model has demonstrated the dependence of membrane excitability on synaptic input conveyed by VIP and GABA, and predicted reduced neural firing and *Per* mRNA oscillation amplitudes as well as shorter circadian periods with reduced cytosolic Ca^2+^ concentrations. These results suggest a dual effect of signaling pathways instigated by VIP and calcium that potentially operate as coupling agents between the gene regulatory network and the electrophysiology of SCN neurons.

## Results

### Oscillatory Profiles of Individual Cellular Clocks

**Figure 1:**
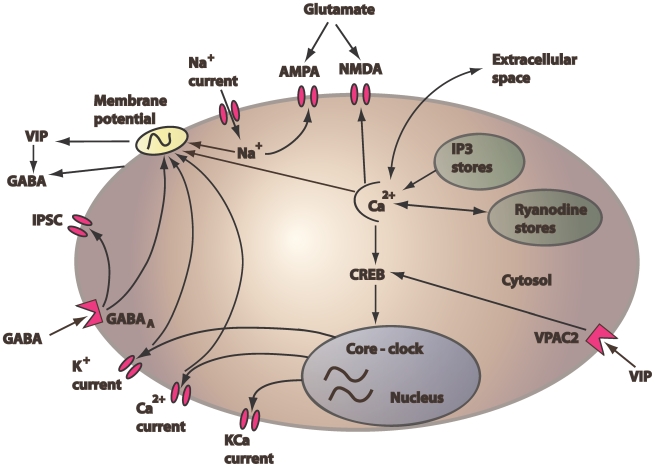
Schematic representation of the SCN neuron model. The gene expression model was obtained from a published study by Leloup and Goldbeter (2003), whereas the intracellular calcium model was adapted from Goldbeter et. al (1990). VIP expressed as a function of firing frequency was responsible for the rhythmic release of GABA. Because our model describes a single SCN cell, we assumed that the VIP and GABA concentrations acting on the cell membrane were the same as the released concentrations. In that sense our model assumes autocrine responses. The signaling cascade that activates Per transcription was adapted from To et. al (2007) to include the effects of intracellular calcium. Extracellular post-synaptic currents involve AMPA and NMDA receptors activated in a constant phase relationship to the Na^+^ and Ca^2+^ concentrations, respectively.

**Figure 2:**
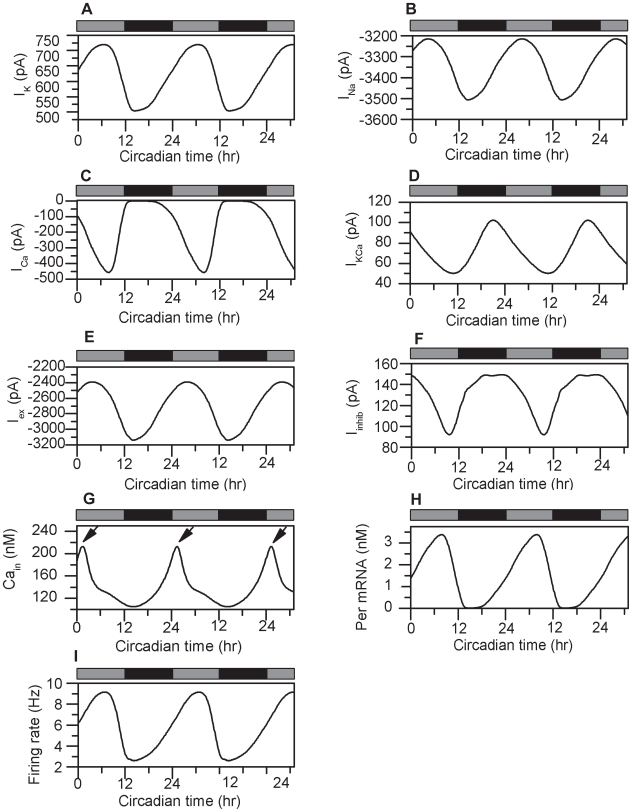
Oscillatory profiles of individual cellular clocks. Rhythmic profiles of the potassium (A), sodium (B), calcium (C), Ca^2+^-activated potassium (D), excitatory (E) and inhibitory (F) currents. Intracellular calcium (G), Per mRNA (H) and the firing rate (I) also displayed oscillations that peaked during the subjective day. The arrows in Fig. 2G denote CT 1.5, which is the time Ca_in_ peaks. The gray and black bars on the top of each figure represent the alternation between the subjective day (gray) and night (black) that compose a 24h circadian cycle in constant darkness.

In this work we developed a firing rate code model to capture the circadian fluctuations of relevant ion channels as well as 24 hour trends in core-clock gene expression. Individual currents (*I_K_*, *I_Na_*, *I_Ca_*, *I_KCa_*, *I_ex_* and *I_inhib_*) were assumed to interact with the circadian gene regulatory network via signaling pathways that included VIP and Ca^2+^ contributions (Fig. 1). Our model produced daily oscillations in constant darkness with 23.6 hour periodicity in the ionic and synaptic currents (Figs. 2A–2F), intracellular calcium concentration (Fig. 2G), *Per* mRNA expression (Fig. 2H) and neural firing rate (Fig. 2I). These rhythmic profiles constitute the nominal output of our model and will be referred to as the “control.”

To calculate the phase relationships of individual rhythmic signals we regarded the cytosolic calcium peak at CT 1.5 [4] as our reference point and computed the phase differences between the Ca^2+^ peak and the peaks of the various rhythmic profiles. This calibration method was used to produce a well tuned model, where circadian components exhibited rhythmic behavior generally matching experimental data both in their relative phase relationships and oscillation peaks as summarized in Table 1. The circadian trend of the sodium current remains unknown [22], but our model predicted circadian oscillations of *I_Na_* with a peak during the subjective night at CT 14.3 (Fig. 2B).

**Table 1:** Circadian phase of SCN model components relative to a calcium peak at CT 1.5.

Rhythmic Output	Time of Peak (CT)	Time of Peak (CT)	Reference
	Model Results	Experimental Data	
Potassium current	6.4	4–6	[7]
Sodium current	14.3	–	None
Calcium current	8.3	4–8	[6]
BK current	21	20	[8]
Inhibitory current	18.5	11–15	[24]
Excitatory current	14.5	4–10	[42]
Calcium	1.5	1.5	[4]
Per mRNA	7.8	4–8	[49]
Firing rate	6.7	6.5	[17]

### Calcium Dynamics and Circadian Regulation

The effects on calcium dynamics on circadian behavior was first studied by clamping the membrane voltage at a hyperpolarized level while maintaining constant calcium concentrations. As observed experimentally [17], the model neuron produced arrhythmic behavior (results not shown). Next we blocked various mechanisms of intracellular calcium accumulation to investigate their effects on firing rate and rhythmic behavior. In an effort to reproduce experimentally observed trends we specifically eliminated IP3-stores, ryanodine stores and L-type Ca^2+^ currents. Ikeda et. al (2003) [4] showed that IP3-stores did not contribute to calcium oscillations and action potential dynamics. In our model, IP3-blockade was simulated by zeroing the rate of calcium release from InsP_3_-sensitive stores (*v_1_*) and observed to reproduce this data (results not shown), demonstrating circadian dynamics of *Per* gene expression were not affected by cytosolic calcium stores.

**Figure 3:**
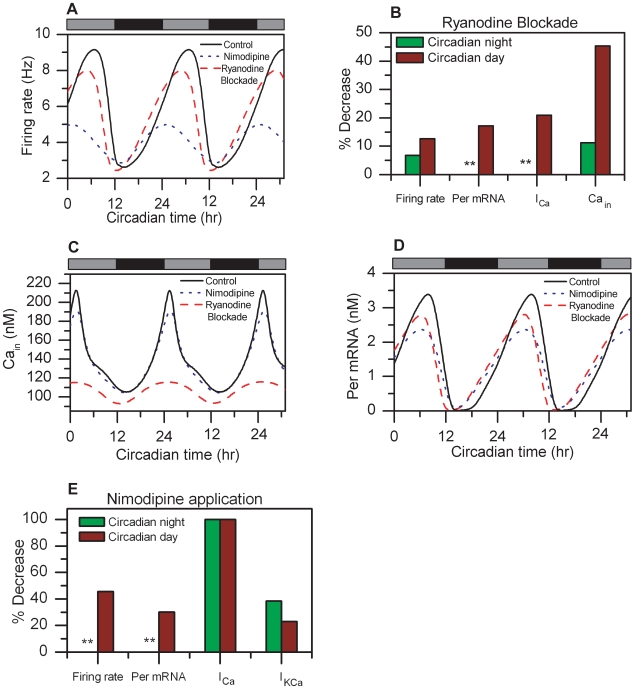
Intracellular calcium dynamics affect the circadian oscillatory behavior. Circadian profiles of the firing rate (A), intracellular calcium concentration (C) and *Per* mRNA concentration (D) are shown for the control (black line), ryanodine blockade (red dashed line) and nimodipine application (blue dotted line). B). % decrease in the firing rate, *Per* mRNA concentration, Ca^2+^ current (*I_Ca_*) and cytosolic calcium concentration during the circadian night (green bars) and day (red bars) as a result of ryanodine blockade. E) % decrease in the firing rate, *Per* mRNA concentration, Ca^2+^ current (*I_Ca_*) and Ca^2+^ -activated K^+^ current (*I_KCa_*) during the circadian night (green bars) and day (red bars) as a result of nimodipine application. ** denotes very small changes of the perturbed value compared to the control.

Ryanodine stores, responsible for Ca^2+^ release into the cytosol, are known to play an important role in the 24h oscillations of the intracellular calcium concentration and action potential frequency [4]. Ryanodine blockade was implemented by zeroing the term that accounts for calcium release from the stores into the cytosol (*v_3_*). Elimination of this term resulted in a decrease in the peak of the neural firing rate by 13% compared to the control during the subjective day (Figs. 3A, 3B), consistent with Ikeda et. al (2003) [4] who reported a 22±8% reduction. Ryanodine blockade had a minimal effect on the firing frequency trough during the subjective night in agreement with experimental findings [4] (Figs. 3A, 3B). Decreases of 11% and 45% in the intracellular calcium trough (subjective night) and peak (subjective day), respectively, were observed compared to the control (Figs. 3B, 3C) consistent with Ikeda et. al (2003) [4].The reduction in intracellular calcium levels had an effect on *I_Ca_*, which was predicted to decrease by 20% during the subjective day. Furthermore, our simulations produced a 17% decrease in the *Per* mRNA amplitude (Fig. 3B, 3D). This trend is consistent with Lundkvist et. al (2005) [20] who demonstrated abolishment of *Per* gene expression rhythms with decreasing intracellular calcium concentrations.

L-type Ca^2+^ currents experimentally blocked via nimodipine application have been shown to affect firing rates, but not intracellular calcium levels, within a single cell [4],[22]. The effects of nimodipine were implemented by setting the L-type Ca^2+^ conductance (*g_Ca_*) to zero. Experimental studies involving nimodipine application were carried out over a maximum period of 5 hours [4], whereas our simulations involved constitutive nimodipine application over the course of the day. Under these conditions the model predicted a slight period decrease of the core-oscillator period to 22.2 h. Simulations of nimodipine application produced a 45% decrease in the firing frequency peak compared to the control during the subjective day, while minimal effects were observed on the minimum firing rate during the subjective night (Figs. 3A, 3E) consistent with experimental studies [4],[22],[23]. Decrease in the intracellular calcium concentration peak by 10% was also observed (Figs. 3C, 3E) in agreement with experiments reporting a 4±6% reduction [4]. The Ca^2+^-activated potassium current (*I_KCa_*) decreased by 23% and 38% during the subjective day and night, respectively (Fig. 3E), in agreement with the literature where 30–50% reductions have been reported [22]. The model produced a reduction of the *Per* mRNA amplitude by 30% (Figs. 3D, 3E), similar to that reported by Lundkvist et. al (2005) [20].

### Effect of GABA on Circadian Rhythmicity

**Figure 4:**
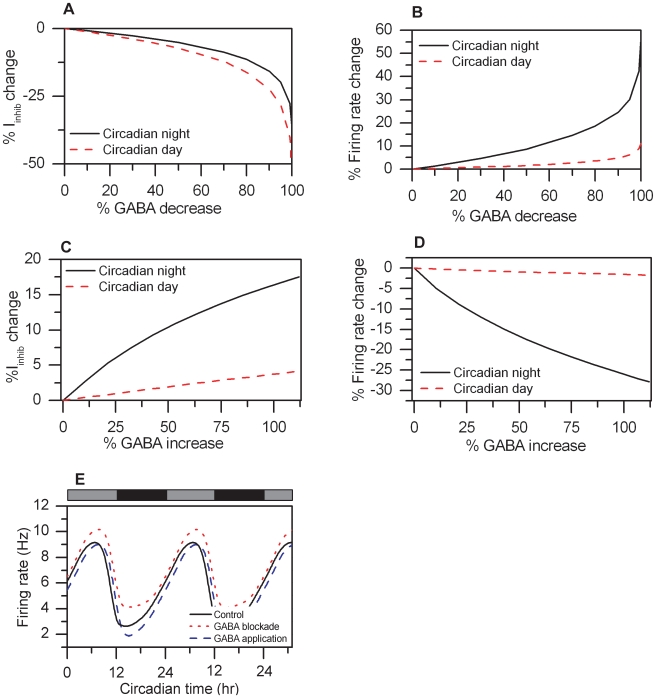
GABA concentrations affect the circadian oscillatory behavior. A). % IPSC change during the subjective night (black solid line) and day (red dashed line) as a function of % GABA decrease B). % firing frequency change during the subjective night (black solid line) and day (red dashed line) as a function of % GABA decrease. C) % IPSC change during the subjective night (black solid line) and day (red dashed line) as a function of % GABA increase. D). % firing frequency change during subjective night (black solid line) and day (red dashed line) as a function of % GABA increase. E). Circadian profiles of the firing frequency are shown for the control (black line), complete GABA blockade (red dotted line) and maximum GABA application (blue dashed line).

We simulated autocrine response of the GABA neurotransmitter to investigate the role of inhibitory postsynaptic currents (IPSCs) on single cell neural firing and circadian behavior. Our initial objective was to simulate the effects of incrementally decreasing GABA concentrations by imposing step reductions in the mean cytosolic GABA level (Eq. 20). The effect of GABA reduction in our system during the subjective day and night was evaluated by computing the percent changes in firing frequency peak and trough versus the control. IPSC levels exhibited dose dependent reductions (Fig. 4A) leading to increased membrane excitability and therefore increased neural firing rate (Fig. 4B, 4E) as GABA concentrations decreased, in agreement with the literature [15]. Consistent with experimental data by Gribkoff et. al (2003) [14] complete blockade of GABA was seen to increase the firing rate peak and trough by 11% and 57%, respectively (Fig. 4E).

Additional simulations involved incremental increase of the GABA concentration binding to the cell surface, implemented by including an additive term in the mathematical expression of GABA (Eq. 20). Our model produced a gradual increase in IPSC levels accompanied by a dose-dependent decrease in neural firing as increasing GABA concentrations were applied (Figs. 4C, 4D), consistent with experimental findings [14]. GABA application was shown to have a minimal effect on the neural firing rate during the subjective day (less than 2%) while significantly decreasing the firing frequency by a maximum of 28% during the subjective night (Fig 4E). This dependence of cellular response on the circadian time of GABA administration has also been demonstrated in experimental studies [14]. Our simulations produced reduced firing frequencies during the subjective night comparable to experimental data. The smaller responses produced during the subjective day, however, did not agree with available data [14].

### Effect of VIP on Circadian Rhythmicity

**Figure 5:**
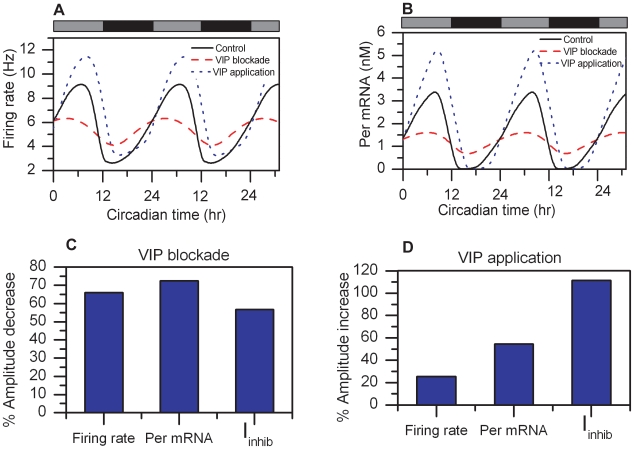
Effects of VIP on circadian rhythmicity. Circadian profiles of firing rate (A) and *Per* mRNA (B) for the control (black line), VIP blockade (red dashed line) and VIP application (blue dotted line). C) % amplitude decrease in firing rate, *Per* mRNA and IPSCs as a result of VIP blockade. D) % amplitude increase in firing rate, *Pe*r mRNA and IPSCs as a result of VIP application.

We simulated autocrine response of the VIP neurotransmitter to investigate the effects of the VIP signaling pathway on single cell behavior. Initially, we zeroed the VIP concentration responsible for the GABA oscillations (Eq. 20) and CREB activation (Eq. 30). Complete VIP blockade reduced firing rate amplitudes by 66% (Figs. 5A, 5C) in agreement with Brown et. al (2007) who reported a 52% average reduction [17]. Our simulations showed an acute decrease in *Per* mRNA levels that oscillated with much lower amplitudes compared to the control (73% decrease; Fig. 5B, 5C), as observed experimentally by Maywood et. al (2006) [16]. Because VIP blockade affects GABA release a 57% reduction in IPSC amplitude was also observed (Fig. 5C), consistent with data from Itri et. al (2004) [24]. VIP elimination resulted in a period decrease to 22.2 h, consistent with Brown et. al (2007) [17] who measured a 22.9±1.9 h period across VIP^−^/^−^ SCN populations.

Additional simulations involved increase of the VIP concentration binding to the cell surface to investigate the effects of constitutive VIP application throughout the circadian cycle. Simulation of VIP application was implemented by adding 1nM to the VIP concentration responsible for GABA release (Eq. 20) and CREB activation (Eq. 30), leading to saturation of the VPAC2 receptors. To our knowledge, experimental data on constant VIP administration throughout a 24 hour period are not currently available. Our simulations showed a 25% increase in the circadian amplitude of the firing frequency (Fig 5A, 5D). Because *Per* gene expression is the final target of the VIP signaling cascade [25], the imposed VPAC2 receptor saturation resulted in increased *Per* mRNA amplitudes of approximately 54% (Figs. 5B, 5D). IPSC amplitudes were observed to increase by 110% (Figs. 5D), comparable to experimental studies [26]. The simultaneous increase in neural spiking and IPSCs can be attributed to the dominant effects of *Per* gene dynamics within the network. Constitutive VIP application decreased the predicted period to 22.5h. These model predictions can be tested experimentally by applying constant VIP concentration throughout a 24 hour period while conducting bioluminescence recordings to measure *Per* gene activation and utilizing multielectrode arrays on highly dispersed SCN cultures to measure firing activity of single cells.

### Intracellular Calcium Concentration as the Circadian Coordinator

**Figure 6:**
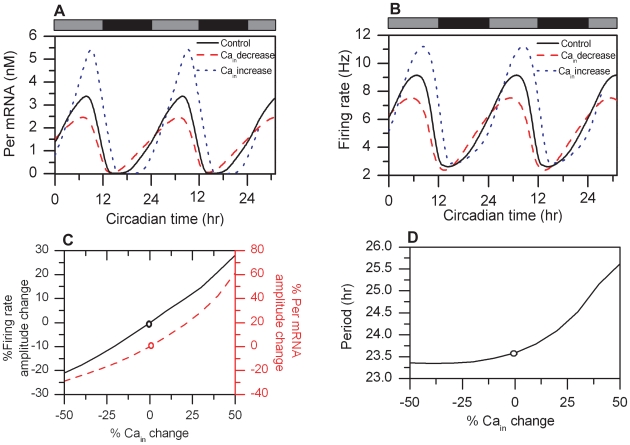
Cytosolic calcium levels regulate circadian behavior. Circadian profiles of *Per* mRNA (A) and firing rate (B) are shown for the control (black line), 50% reduced cytosolic Ca^2+^ concentration (red dashed line) and 50% increased cytosolic Ca^2+^ concentration (blue dotted line) compared to the control. C). *Per* mRNA (red dashed line) and firing rate (black solid line) amplitudes as a function of the cytosolic calcium concentration. D). The period of the core oscillator as a function of the cytosolic calcium concentration. The circles in 6C and 6D represent nominal values of the model.

We varied the intracellular calcium concentration to investigate its effects on the rhythmic output of the circadian clock. Changes in effective Ca^2+^ levels were achieved by scaling the output of Eq. 12, responsible for the circadian evolution of intracellular calcium, by multiplying with a scaling factor ranging from 0.5 to 1.5. Hence mean levels of calcium were varied by ±50% of their nominal value. Because our model was constructed under the assumption that cytosolic calcium instigates a signaling cascade with *Per* gene transcription as the final product [19] incrementally increasing Ca^2+^ concentrations had a positive effect on *Per* mRNA amplitudes (Figs. 6A, 6C). A similar trend was observed for neural firing, as increasing intracellular calcium increased firing frequency amplitudes (Figs. 6B, 6C). The calcium concentration also affected the periodicity of the model system. Increased Ca^2+^ levels produced longer periods of the core oscillator, reaching a maximum of 25.6h for a 50% Ca^2+^ increase (Fig. 6B).

Our model predictions can be compared with experimental data on SCN explants, which show abolished mean *Per* mRNA rhythms when averaged over the entire population as a function of increasing concentrations of Ca^2+^ buffer [20]. Our simulations suggest that the observed elimination of collective *Per* gene expression rhythm across the population can be attributed to reductions in the amplitude of individual *Per* mRNA signals accompanied by a period decrease on the single cell level. This hypothesis requires further experimental studies for validation.

### Correlating Electrophysiology with Gene Expression

**Figure 7:**
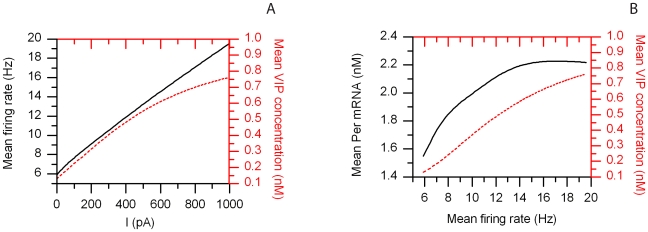
Correlating electrophysiology with gene expression. A). Mean firing rate (black solid line) and mean VIP concentration (red dashed line) as a function of the applied extracellular current (*I*). B). Mean VIP concentration (red dashed line) and mean *Per* mRNA levels (black solid line) versus the mean firing rate.

Incrementally increasing current levels were applied on the cell membrane of our neuron model to investigate the effects of extracellular electrical stimuli on single cell behavior. Current application was implemented by including an additive term in the mathematical expression of I^*^ (Eq. 3). As expected, a positive, linear correlation of firing rate with inward current levels (*I*) was observed (Fig 7A). Because VIP is released as a function of firing rate (Eq. 29), VIP concentrations were also seen to increase with electrical stimuli (Fig. 7A). The direct relationship between mean firing rate and mean VIP concentration over the course of a circadian cycle is shown in Fig 7B. Increasing electrical stimuli did not significantly contribute to mean intracellular calcium levels (results not shown). Neural firing was predicted to correlate with core-clock gene transcriptional activity as demonstrated by Quintero et. al (2003) [11]. Mean *Per* mRNA levels were predicted to increase as a function of the mean neuronal spiking frequency and ultimately obtained their maximum after a threshold in firing rate had been reached (Fig 7B).

Our model demonstrated a positive relationship between electrical firing and core-clock gene activity consistent with the literature [11],[12]. Simulations of increasing electrical stimuli suggest correlations between *Per* mRNA levels and the firing frequency potentially mediated via a VIP-instigated signaling pathway. Our model postulates an underlying intracellular network where VIP, released due to elevated neuronal spiking, binds on the cell surface initiating a signaling mechanism that leads to *Period* gene activation.

## Discussion

We developed a multiscale mathematical model to investigate the association between the circadian gene regulatory pathway, electrophysiology and cytosolic calcium and to evaluate SCN single cell behavior over a circadian time scale. Initially, we investigated the effects of calcium dynamics on cell behavior by simulating the blockade of L-type Ca^2+^ channels, IP3- and ryanodine stores. The model successfully replicated a number of experimental observations. The effect of synaptic input on membrane excitability was explored by assuming autocrine response of the VIP and GABA neurotransmitters. Simulations of variable GABA concentrations and VIP blockade were conducted to compare the model output with experimental data and test model validity. Increasing the GABA concentration was shown to have an inhibitory effect on neural firing, matching experimental data. Several experimental studies have reported excitatory responses in a subset of SCN neurons depending on the circadian timing of GABA administration [27],[28]. These effects have not been included in the present model but will be considered in our future work. Simulations of VIP blockade produced decreased amplitudes in firing frequency, *Per* mRNA and IPSCs compared to the control, all in agreement with the literature. We further tested the effects of constitutive VIP application, for which experimental data are not currently available. Our model predicted increased amplitudes in *Per* mRNA, firing rate and IPSCs compared to the control as VPAC2 receptors became saturated.

Our model postulates that calcium plays an important role in the coordination of neural firing and core clock gene expression. To test this hypothesis, we gradually altered the intracellular Ca^2+^ concentration and determined the effect on single-cell rhythmic behavior. Amplitude reductions in *Per* mRNA and neural firing accompanied by a period decrease were observed as the cytosolic Ca^2+^ concentration was gradually reduced. Experimental data showing the role of cytosolic Ca^2+^ on circadian behavior are currently available only for SCN explants [20]. These data showed elimination of the mean *Per* mRNA rhythm, averaged over the entire population, as cytosolic Ca^2+^ was gradually buffered. Thus, our simulations suggest that reduction in the amplitude of individual *Per* mRNA signals accompanied by a period decrease and deregulation of the phase relationships between the various circadian components may be manifested experimentally as the elimination of the collective *Per* gene expression rhythm across the population. This hypothesis can be tested experimentally by adding a buffer (e.g. the intracellular chelator BAPTA-AM) to reduce cytosolic Ca^2+^ while conducting bioluminescence recordings to measure *Per* gene activity of a single cell and utilizing multielectrode arrays on highly dispersed SCN cultures to measure neural firing activity.

Our model demonstrated a positive correlation of core-clock gene expression with the neural spike frequency consistent with the literature [11],[12]. Incrementally increasing electrical stimuli applied on the cell membrane of our neuron model affected the mean levels of the firing rate, *Per* gene expression and VIP release. Furthermore positive relationships between VIP release and core-clock gene transcription with firing rate were observed. We hypothesized an underlying intracellular network where VIP, released due to elevated neuronal spiking, binds on the cell surface initiating a signaling cascade that leads to *Per* gene activation.

Our single cell model was shown to replicate a number of experimentally observed trends, as well as to provide predictions concerning intracellular couplings. One of the key features of the model is the ability to test effects of blockers, neurotransmitters or extracellular stimuli for prolonged periods of time, which can be challenging in an experimental setup. Long-term application of ryanodine blockers or intracellular calcium buffers (BAPTA-AM), for example, is known to disrupt intracellular Ca^2+^ from physiological levels [4],[20]. A number of vital cell processes depend on calcium activity, rendering the interpretation of such experiments in terms of a single effect on the circadian network difficult. The model may therefore provide predictions that assist in the development of carefully designed experiments to test these hypotheses.

## Materials and Methods

### Intracellular Oscillator Model

The core oscillator utilized in our model originates from a previous study [29] and consists of 16 ordinary differential equations in time that describe intertwined negative and positive regulatory transcriptional loops. Transcription of the *Per* and *Cry* genes is activated by a heterodimer formed from the CLOCK and BMAL1 proteins. This activation is rhythmically suppressed and reestablished by a complex of the PER and CRY proteins, which blocks the activity of CLOCK/BMAL1 dimer and negatively autoregulates transcription of the *Per* and *Cry* genes. Our model did not include the loop involving *Rev-Erbα* since it was not required for sustained circadian oscillations. Nominal parameters values were mostly obtained from the original reference, with the exception of the parameter *k*_1_ that determines the transport rate of the PER/CRY complex from the cytosol to the nucleus, *K_AP_* involved in *Per* activation due to elevated BMAL1 concentrations and *v_sp0_* that denotes the basal value of *Per* transcription rate. These values were modified as part of the model tuning process (see Table 2).

**Table 2:** Model parameter values.

Parameter	Value	Reference	Parameter	Value	Reference
*θ*	(20 + V_rest_) mV	[22]	*v_Cl1_*	15.5 mM	
*E_K_*	−97mV	[35]	*v_Cl2_*	19 mM	
*T*	37°C		*K_Cl1_*	4 nM	
*g_Ko_*	9.7 nS		*K_Cl2_*	1 nM^−0.2^	
*v_gk_*	10 nS		*n_Cl_*	−0.2	
*K_gk_*	10 nM		*Cl_ex_*	114.5 mM	[40],[41]
g_Na_	36 nS	[22]	v_ex1_	105 nS	
E_Na_	45mV	[22]	*K_ex1_*	574.05 µA^2.5^	
*v_kk_*	3.3 µM ^−1^ h^−1^		*n_ex1_*	2.5	
*K_kk_*	0.02 nM ^0.1^		*v_ex2_*	4.4 nS	
*nkk*	0.1		*K_ex2_*	1 µM ^−1^	
*v_vo_*	0.09 µM h^−1^		*n_ex2_*	−1	
*K_vo_*	4.5 nM ^4.5^		*E_ex_*	0 mV	[30]
*nvo*	4.5		*P_Ca_*	0.05	[39],[47]
*v*	2		*P_Na_*	0.036	[39],[47]
*v_1_*	0.0003 µM h^−1^	[21] *	*P_Cl_*	0.3	[39],[47]
*β_IP3_*	0.5	[21] *	*K_ex_*	1 mM	[39],[47]
*V_M2_*	149.5 µM h^−1^	[21] *	*Na_ex_*	145 mM	[39],[47]
*K_2_*	5 µM	[21] *	*v_PK_*	1.9	
*n*	2.2	[21] *	*K_PK_*	1 nM^−2^	
*V_M3_*	400 µM h^−1^	[21] *	*npk*	−2	
*K_R_*	3 µM	[21] *	*V_R_*	0.41 GΩ	
*m*	6	[21] *	*K_R_*	34 mV	
*K_A_*	0.67 µM	[21] *	*v_VIP_*	0.5 nM h^−1^	
*p*	4.2	[21] *	*K_VIP_*	15 Hz^1.9^	
*k_f_*	0.001 h^−1^	[21] *	*n_VIP_*	1.9	
*Ca_ex_*	5 µM		*k_dVIP_*	0.5 nM^0.8^ h^−1^	
*v_Ca_*	12.3 nS		*n_dVIP_*	0.2	
*K_Ca_*	22 nM^2.2^		*V_MK_*	5 nM h^−1^	
*n_Ca_*	2.2		*K_MK_*	2.9 µM	
*v_KCa_*	3 nS		*V_β_*	2 nM h^−1^	[48] *
*K_KCa_*	0.16 nM^−1^		*K_β_*	2	[48] *
*n_KCa_*	−1		*C_T_*	1.6 nM h^−1^	[48] *
E_L_	−29 mV	[22]	*K_c_*	0.15 nM	[48] *
*GABA_o_*	0.2 nM		*K_D_*	0.08 nM	[48] *
*v_GABA_*	19 nM		*k_1_*	0.45 h^−1^	[29] *
*K_GABA_*	3 nM		*K_AP_*	0.6 nM	[29] *
*g_GABA_*	12.3 nS	[40]	*v_sp0_*	1 nM h^−1^	[29] *
*Cl_o_*	1 mM		*C_m_*	5 nF	
			*V_reset_*	(4 + V_rest_) mV	

*These parameter values were altered from the values in the original references as part of the model tuning process.

### Electrophysiology Model

In this work we utilized a modified integrate-and-fire model [30] that takes into account the contributions of the relevant ion channels and includes the effects of extracellular synaptic stimuli. Following the methodology of Liu and Wang (2001) [31] we constructed a SCN membrane model that included sodium (*I_Na_*), potassium (*I_K_*), calcium (*I_Ca_*) and calcium-activated potassium (*I_KCa_*) currents, as proposed by Brown et al (2007) [3]. The contributions of inhibitory and excitatory input signals, known to influence membrane excitability, were also incorporated in our model. Individual ionic and synaptic currents (*I_r_*) were modeled as:
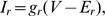
(1)where *V* represents the membrane voltage, *g_r_* is the conductance and *E_r_* is the reversal potential of current *r*.

Our single SCN model neuron was described by a modified integrate-and-fire model [31]–[34]:
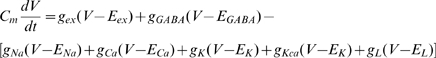
(2)where *C_m_* denotes the membrane capacitance and *g_ex_*, *g_GABA_*, *g_Na_*, *g_Ca_*, *g_Κ_*, *g_ΚCa_*, and *g_L_* are the conductances for the excitatory, inhibitory, sodium, calcium, potassium, calcium-activated potassium and leakage currents, respectively, and *E_ex_*, *E_GABA_*, *E_Na_*, *E_Ca_*, *E_Κ_*, and *E_L_* are the corresponding reversal potentials. Activation and inactivation variables associated with the relevant conductances were not included in this model (Eq. 2) as they evolve on the millisecond timescale rather than the circadian timescale considered in the study.

By defining:
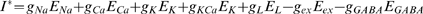
(3)

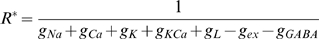
(4)

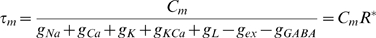
(5)Eq. (2) can be reformulated to yield [30]: 
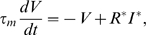
(6)


The integrate-and-fire model does not produce the form and shape of an action potential. Rather neural spikes can be characterized by a “firing time” *t_f_*, defined by the criterion:
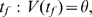
(7)where *θ* is the firing threshold. Immediately after t_f_ the potential is reset to a new value *V_reset_*<*θ*. To calculate the trajectory of the membrane potential after the occurrence of a spike at time t_f_, Eq. 6 was integrated with the initial condition *V(t_(1)_) = V_reset_*. Because time variations in I^*^, R^*^and *τ_m_* were much faster than the circadian timescale they were considered constant for each simulated 10 minute time step. Therefore integration of Eq. 6 yields:
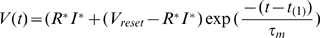
(8)The membrane potential described by Eq. 8 approaches the asymptotic value V(∞) = R^*^I^*^ as t→ ∞. Therefore since R^*^I^*^>*θ* the membrane potential reaches the threshold *θ* at time t_(2)_:
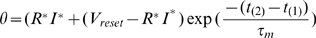
(9)The time interval *t_(2)_*−*t_(1)_* constitutes the firing period *T^′^*. Thus the firing rate (*f_r_*) can be calculated as the inverse of *T^′^* [30], yielding the firing rate code model used in this study: 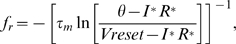
(10)The firing rate (eq. 10) fluctuates due to circadian variations in conductances and reversal potentials of the various currents, which are included within the terms I^*^, R^*^ and *τ_m_* (eqs. 3–5).

#### Potassium current

The model includes the effects of potassium channels, as studied by Bouskila and Dudek [35]. The reversal potential of potassium (*E_K_*) experimentally determined at room temperature (22°C) [35] was adapted for 37°C (body temperature) in all our simulations by multiplying with the body-to-room temperature ratio. This mathematical correction was based upon the Nernst equation, which provides the relation between reversal potential (*E_K_*) and temperature [36]. The conductance of potassium channels (*g_K_*) was modeled to oscillate in 24 hour cycles and peak during the circadian day as found experimentally [7]. 
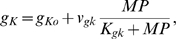
(11)where g_Ko_ denotes the basal value of potassium conductance, *v_gk_* the maximum rate, *K_gk_* the saturation constant of potassium channel dynamics and *MP* the *Per* mRNA concentration. Eq. 11 was not intended to imply a mechanistic relationship between *g_K_* and *MP* but instead to yield experimentally observed circadian behavior.

#### Sodium current

Sodium current dynamics were incorporated in our model using data from Jackson et al. [22]. The values of sodium conductance (*g_Na_*) and reversal potential (*E_Na_*) were obtained from [22] and were corrected for 37°C as described above.

#### Calcium current

The model included L-type calcium currents, as studied by Pennartz et al. [6]. The calcium reversal potential (*E_Ca_*), calculated via the Nernst equation, oscillated within a physiological range [22],[37]. The cytosolic calcium concentration (*Ca*) was modeled to oscillate over a 24 hour period and peak during the subjective day, consistent with findings of Ikeda et al. [4]. The intracellular calcium model utilized was adapted from a previous study [21] and included the bidirectional flow of Ca^2+^ ions through the cell membrane, as well as the effects of IP3- and ryanodine stores: 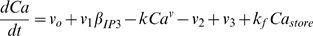
(12)

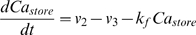
(13)In these equations *k* represents the efflux of calcium out of the cell and *v_o_* is the influx of calcium into the cytosol. These effects have been altered from the original reference [20], where they were regarded constant, to account for daily variations of Ca^2+^ flux through various Ca^2+^ ion channels as well as passive transport. The calcium efflux was modeled as:
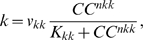
(14)where *v_kk_* is the maximum rate, *K_kk_* is the saturation constant, *nkk* is the cooperativity coefficient of calcium efflux dynamics, and *CC* denotes the cytosolic, unphosphorylated CRY protein concentration. Eq. 14 was not intended to imply a mechanistic relationship between *k* and *CC*, but instead was introduced to yield experimentally observed circadian dependent behavior.

The calcium influx was modeled as:
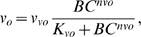
(15)where *v_vo_* is the maximum rate, *K_vo_* is the saturation constant, *nvo* is the cooperativity coefficient of the calcium influx dynamics, and *BC* denotes the unphosphorylated, cytosolic BMAL1 protein concentration. As before, Eq. 15 was not intended to imply a mechanistic relationship between *v_o_* and *BC*. The release of calcium from InsP_3_-sensitive stores was controlled by *v_1_* and the *β_IP3_*, both of which were regarded constant for our simulations. The rate constant for leaky release of calcium from the ryanodine pool (*k_f_*) was also considered constant. Detailed descriptions of *v_2_*, the transport of calcium from the cytosol to the ryanodine stores, and *v_3_*, the release of calcium from the stores into the cytosol, were obtained from the original reference [21]:
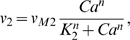
(16)

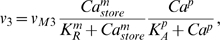
(17)where *V_M2_* and *V_M3_* denote the maximum rates of Ca^2+^ pumping and release from the intracellular store; *K_2_*, *K_R_* and *K_A_* are the threshold constants for pumping, release and activation; *m*, *n*, *p* denote the cooperativity coefficients of these processes. Parameter values utilized in the *v_2_* and *v_3_* expressions have been altered from the original study as part of the model tuning process (see Table 2). The conductance of the Ca^2+^ channels (*g_Ca_*) was rhythmically altered throughout the circadian cycle and peaked during the subjective day [22]:
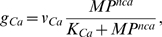
(18)where *v_Ca_* is the maximum rate, *K_Ca_* the saturation constant of calcium channel dynamics and *nca* the cooperativity coefficient. As before, Eq. 18 was not intended to imply a mechanistic relationship between *g_Ca_* and *MP*.

#### Calcium-activated potassium current

Our model incorporated the effects of large-conductance Ca^2+^-activated potassium (BK) currents as studied by Meredith et al. [8] and Pitts et al. [9]. We modeled the conductance of the BK channels (*g_KCa_*) to oscillate over the course of the day and to peak during subjective night consistent with Pitts et al. [9].
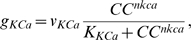
(19)where *v_KCa_* is the maximum rate, *K_KCa_* is the saturation constant of Ca^2+^-activated K^+^ channel dynamics and *nkca* denotes the cooperativity coefficient. Eq. 19 was not intended to imply a mechanistic relationship between *g_KCa_* and *CC*.

#### Leakage current

Leakage currents have been included in the model to account for the natural permeability of the membrane and the passive transport of ions in and out of the cell. The resting potential (*E_L_*) was obtained from Jackson et al. [22] and corrected for a temperature of 37°C. The conductance was *g_L_* = 1/*R* [37], where *R* denotes the membrane resistance, described in detail below.

#### Inhibitory current

Our model included the effects of inhibitory postsynaptic currents (IPSCs), conveyed by the GABA neurotransmitter and its GABA_A_ receptor. GABA was rhythmically released from the cell as a function of VIP in agreement with the finding of Itri and Colwell [24],[26]:
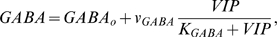
(20)where *GABA_o_* denotes the basal value, *v_GABA_* the maximum rate and *K_GABA_* the saturation constant of GABA oscillations. Because our simulations involved single SCN cells, the GABA concentration binding on the membrane surface of our model neuron was assumed to be equal to the GABA concentration released by the neuron (Eq. 20). In this sense our model has assumed an autocrine response of GABA, selectively activating Cl^−^ channels on the cell membrane and causing Cl^−^ influx into the cytosol (Fig. 1). Our model included sustained 24h fluctuations in the intracellular Cl^−^ concentration (*Cl_in_*) that peaked during the subjective day in agreement with Wagner et al. [38] and were further amplified as a function of GABA:
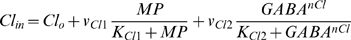
(21)where *Cl_o_* denotes the basal intracellular Cl^−^ concentration, *v_C1l_* and *K_Cl1_* denote the maximum rate and the saturation constant of PER controlled Cl^−^ release into the cytosol, v*_Cl2_* and *K_Cl2_* denote the maximum rate and the saturation constant of GABA induced Cl^−^ release into the cytosol, and *n_Cl_* represents the cooperativity coefficient. The extracellular Cl^−^ concentration (*Cl_ex_*) was obtained from previous studies [39],[40] to yield inhibitory reversal potentials (*E_GABA_*) that oscillated within a physiological range [40],[41]. The value of IPSC conductance (*g_GABA_*) was obtained from the literature [40].

#### Excitatory current

The contributions of excitatory postsynaptic currents (EPSCs), typically observed in response to the glutamate neurotransmitter, were incorporated in our model. Glutamate is expressed by the ganglion cells of the retinohypothamalic tract (RHT) that project to the SCN and is also present in all neurons as part of the normal metabolic pool of amino acids, rendering its distinction from the neurotransmitter pool difficult. Circadian variations in EPSCs have been shown within the SCN and have been correlated with diurnal fluctuations in AMPA receptor activation [42],[43], i.e periodic Na^+2^ influx, as well as NMDA-evoked Ca^2+^ transients [44],[45]. Therefore, the conductance of the excitatory current (*g_ex_*) was modeled to oscillate in a constant phase relationship to *I_Na_* and *Ca*.
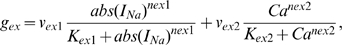
(22)where v*_ex1_* and *K_ex1_* represent the maximum rate and saturation constant of AMPA- induced EPSCs; v*_ex2_* and *K_ex2_* represent the maximum rate and saturation constant of NMDA-induced EPSCs; *n_ex1_* and *n_ex2_* are cooperativity coefficients. The reversal potential of the excitatory synaptic current (*E_ex_*) was assumed to be constant consistent with the literature [30].

### Membrane Properties

Membrane properties such as the resting potential and resistance display sustained circadian rhythms [5],[6]. Our model included oscillations of the membrane resting potential (*V_rest_*) by utilizing a modified version of the Goldman-Hodgkin-Katz equation derived by Piek (1975) [46] that takes into account both monovalent ions and divalent ions, such as Ca^2+^:
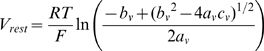
(23)

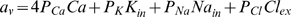
(24)

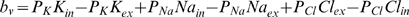
(25)

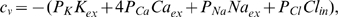
(26)where *R* denotes the gas constant, *F* is the Faraday constant, *P_Ca_*, *P_K_*, *P_Na_*, and *P_Cl_* are the membrane permeabilities of Ca^2+^, K^+^, Na^+^ and Cl^−^, respectively, *K_in_* and *Na_in_* represent the K^+^ and Na^+^ concentrations within the cytosol, whereas *K_ex_*, Ca_ex_ and *Na_ex_* are the K^+^, Ca^2+^ and Na^+^ concentrations in the extracellular space. Values for *P_Ca_*, *P_Na_*, *P_Cl_*, *K_ex_*, *Cl_ex_* and *Na_ex_* were chosen to match experimental measurements from the literature [39],[47], whereas *K_in_* and *Na_in_* were computed by inversion of the Nernst equation. *P_K_* values were modeled to vary over the course of the day in agreement with Kuhlman et. al. [5], who demonstrated circadian rhythmicity in K^+^ currents underlying the membrane potential oscillations:
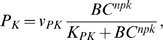
(27)where *v_PK_* is the maximum value, *K_PK_* is the saturation constant and *npk* is the cooperativity coefficient of the P_K_ oscillations. Eq. 27 was not intended to imply a mechanistic relationship between *P_K_* and *BC*.

We modeled the membrane potential to oscillate over the course of the day and to peak during the subjective day. The membrane resistance (*R*) oscillated in a constant phase relationship with the resting potential and peaked during the subjective day as shown by experimental studies [5],[6]:
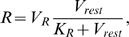
(28)where *V_R_* represents the maximum value and *K_R_* the saturation constant of the membrane resistance oscillations.

### Intracellular Pathways

Core clock gene transcription displays self-sustained circadian rhythms that are likely modulated via VIP/VPAC2 activation [25] and fluctuations in intracellular calcium dynamics [19](Fig. 1). We utilized a revised signaling transduction mechanism from our previous study [48] to capture the effects of these two components on gene regulation. In this study, VIP oscillations were assumed to depend on the neural spike frequency as well as the rate governing the depletion of the neurotransmitter from the synaptic cleft:
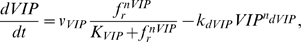
(29)where *v_VIP_* is the maximum rate, *K_VIP_* the saturation constant and *nVIP* the cooperative coefficient of VIP release, while *k_dVIP_* denotes the rate constant and *n_dVIP_* the cooperativity coefficient of VIP depletion. The VIP concentration binding on the membrane surface of our model neuron was assumed to be equal to the VIP concentration released by the neuron (Eq. 20), consistent with an autocrine response.

The intracellular calcium concentration also displayed rhythmic variations over the course of the day (Eqs. 12–13). The transduction mechanism involving the VPAC2 receptor and Ca^2+^ likely includes the activation of protein kinases, which in turn phosphorylate CREB, leading to core clock gene activation. Protein kinase activity was modeled as: 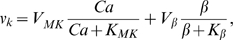
(30)where *v_k_* is the rate of kinase activity, *V_MK_* and *V_β_* denote the maximum rates of Ca^2+^- and VIP-induced protein kinase activation, respectively, and *K_MK_* and *K_β_* represent the saturation constants of Ca^2+^- and VIP-induced protein kinase activation, respectively. Circadian fluctuations in the phosphorylated CREB fraction, as well as the dynamics of the *Per* gene activation, are described in detail in our original study [48]. Parameter values altered from the original reference are displayed in Table 2.

### Simulations and Analysis

The complete single cell model was formulated within MATLAB (The MathWorks, Natick, MA) and consisted of twenty ordinary differential equations (ODEs). Sixteen ODEs described the circadian evolution of the gene transcriptional loop (for details refer to [29]), two ODEs described intracellular calcium rhythms (Eqs. 12–13 modified from [21]), and the remaining two ODEs described the VIP concentration (Eq. 29) and the phosphorylated CREB concentration (for details refer to [48]). The model was integrated numerically using the differential-algebraic equation solver ode23 with a 10 minute time step to ensure accurate solutions with reasonable computational cost. Nominal parameter values utilized in our model are listed in Table 2, with parameters directly obtained from the literature accompanied by the corresponding reference. The tuning of the remaining parameters is discussed in detail below.

- Nominal values for the parameters *g_Ko_*, v*_gk_* and *K_gk_* utilized in Eq. 11 were selected to produce 24 hour oscillations in *g_K_* with a mean value of 11.3nS and a standard deviation of ±1.8 nS, matching experimental data [35].
- The parameters utilized in the intracellular calcium model, *v_kk_*, *K_kk_*, *n_kk_*, *v_vo_*, *K_vo_*, *nvo*, *v_1_*, *β_IP3_*, *k*, *V_M2_*, *K_2_*, *n*, *V_M3_*, *K_R_*, *m*, *K_A_*, *p* and *k_f_*, (Eqs. 12–17) were adjusted from values in the original reference [21] to produce ∼24 hour oscillations in *Ca* that peaked during the subjective day. Intracellular calcium concentrations were predicted to be ∼100% higher during the day compared to the subjective night, consistent with data from Ikeda et al. [4]. The extracellular calcium concentration, *Ca_ex_*, was set at 5 mM to yield calcium reversal potentials, *E_Ca_*, that oscillated in the range of 50–70 mV, as shown in the literature [22],[37]. Parameters used for the calculation of the L-type calcium channel conductance, *g_Ca_*, (Eq. 18), including v*_Ca_*, K*_Ca_* and n*_Ca_* were adjusted to produce oscillations in the range of 0.3≤*g_Ca_*≤1.9 nS as shown experimentally [22].
- Parameters v*_Kca_*, *K_KCa_* and n*_KCa_* utilized for the calculation of the BK channel conductance, *g_Kca_* (Eq.19), were adjusted to produce oscillations in the range 1.5≤g_KCa_≤3.6 nS that peaked during the subjective night, consistent with Pitts et. al [9].
- The parameters *GABA_o_*, *v_GABA_*, *K_GABA_*, *Cl_o_*, *v_C1l_*, *K_Cl1_*, *v_Cl2_* and *K_Cl2_* and n*_Cl_* were utilized for the simulation of IPSC dynamics (Eqs. 20–21). Nominal values for these parameters were selected to: a) produce 24 h oscillations in *Cl_in_* that ranged from 11 to 19 mM and peaked during the subjective day [41] and b) generate inhibitory postsynaptic currents that peaked during the subjective night [24]. The extracellular Cl^−^ concentration was set at *Cl_ex_* = 114.5 mM [40] to yield inhibitory reversal potentials, *E_GABA_*, that oscillated within the range 50–70 mV, consistent with the literature [40],[41].
- The parameters v*_ex1_*, K*_ex1_*, *n_ex1_*, v*_ex2_*, K*_ex2_*, and *n_ex2_* (Eq. 22) were found to have an effect on the firing frequency, *f_r_*. Nominal values for these parameters were chosen to produce *f_r_* oscillations within the range 2≤*f_r_*≤9 Hz that peaked during the circadian day, consistent with experiments [6].
- Nominal values for parameters v*_PK_*, *K_PK_* and *npk* utilized in Eq. 27 were selected to produce *P_K_* oscillations with a mean value of 0.5 and a peak during the subjective night, consistent with Kuhlman et al. [5]. *P_K_* oscillations underlying the rhythm in membrane potential (*V_rest_*, Eqs. 23–26) [5],[6] produced values in the range −52≤V_rest_≤−42mV that peaked during the subjective day consistent with the literature [5],[6].
- The parameters *V_R_* and *K_R_* utilized in Eq. 28 for the computation of the membrane resistance (*R*) were adjusted to produce oscillations in the range 1≤R≤2 GΩ that peaked during the subjective day, as shown by Kuhlman et al. [5],[6]. The membrane capacitance, C_m_, was adjusted to produce firing rate oscillations within a physiological range.

The initial conditions utilized in simulations of the 20 ordinary differential equations characterizing our model system are listed in Table 3. These values were chosen to produce individual rhythmic profiles that oscillated within a reasonable range, consistent with experimental data.

**Table 3:** Initial conditions of the 20 ODEs characterizing the single cell model.

Ca	0.10 µM	PC_N_ *	0.16 nM
Ca_store_	0.10 µM	PC_CP_ *	0.20 nM
M_P_ *	2.80 nM	PC_NP_ *	0.091 nM
M_C_ *	2.00 nM	B_C_ *	2.41 nM
M_B_ *	7.94 nM	B_CP_ *	0.48 nM
P_C_ *	0.40 nM	B_N_ *	1.94 nM
C_C_ *	12.0 nM	B_NP_ *	0.32 nM
P_CP_ *	0.13 nM	I_N_ *	0.05 nM
C_CP_ *	9.00 nM	CB**	0.12 nM
PC_C_ *	1.26 nM	VIP	0.00 nM

*: These variables are part of the core-oscillator model by Leloup and Goldbeter (2003) [29].

**: These variables are part of the VIP signaling model by To et al. (2007) [48].
